# Music induces universal emotion-related psychophysiological responses: comparing Canadian listeners to Congolese Pygmies

**DOI:** 10.3389/fpsyg.2014.01341

**Published:** 2015-01-07

**Authors:** Hauke Egermann, Nathalie Fernando, Lorraine Chuen, Stephen McAdams

**Affiliations:** ^1^Centre for Interdisciplinary Research in Music Media and Technology, Schulich School of Music, McGill UniversityMontreal, QC, Canada; ^2^Audio Communication Group, Technische Universität BerlinBerlin, Germany; ^3^Laboratoire de Musicologie Comparée et Anthropologie de la Musique, Faculté de Musique, Université de MontréalMontréal, QC, Canada

**Keywords:** emotion, affect, music, psychophysiology, universal, cross-cultural

## Abstract

Subjective and psychophysiological emotional responses to music from two different cultures were compared within these two cultures. Two identical experiments were conducted: the first in the Congolese rainforest with an isolated population of Mebenzélé Pygmies without any exposure to Western music and culture, the second with a group of Western music listeners, with no experience with Congolese music. Forty Pygmies and 40 Canadians listened in pairs to 19 music excerpts of 29–99 s in duration in random order (eight from the Pygmy population and 11 Western instrumental excerpts). For both groups, emotion components were continuously measured: subjective feeling (using a two- dimensional valence and arousal rating interface), peripheral physiological activation, and facial expression. While Pygmy music was rated as positive and arousing by Pygmies, ratings of Western music by Westerners covered the range from arousing to calming and from positive to negative. Comparing psychophysiological responses to emotional qualities of Pygmy music across participant groups showed no similarities. However, Western stimuli, rated as high and low arousing by Canadians, created similar responses in both participant groups (with high arousal associated with increases in subjective and physiological activation). Several low-level acoustical features of the music presented (tempo, pitch, and timbre) were shown to affect subjective and physiological arousal similarly in both cultures. Results suggest that while the subjective dimension of emotional valence might be mediated by cultural learning, changes in arousal might involve a more basic, universal response to low-level acoustical characteristics of music.

## Introduction

Although music occurs in all human cultures, its structure and function are highly varied (examples include the use of different pitch scales and differences in ceremonial and emotional uses). Despite these differences, there are some basic perceptual and structural universals in music that are observed across cultures. These universals can inform theories about the evolutionary origins of music, as they might indicate innate properties underlying musical behaviors (Eibl-Eibesfeldt, 1979). Examples of perceptual universals include pitch perception, octave generalization, categorical perception of discrete scale pitches, melodic stream segregation, and perception of melodic contour (Harwood, 1976). Previous research also indicates that the communication of emotional expression in music, a more complex phenomenon, may transcend cultural boundaries. The recognition of expressed emotion in music has been described as being based on underlying psychoacoustic cues that are employed in similar fashion across cultures to convey emotion (Balkwill and Thompson, 1999; Balkwill et al., 2004; Laukka et al., 2013; Sievers and Polansky, 2013). A controlled, cross-cultural investigation testing for such innate emotional universals in music requires the comparison of participant responses from different cultures to the same music. Moreover, it would require that participants from one culture be completely naïve to the music of the other culture. It becomes increasingly difficult to find cultures that fulfill this prerequisite due to the homogenizing effects of globalization, as modern digital music distribution networks have caused Western music and culture to spread rapidly across the globe like never before (Huron, 2008). In a cross-cultural study, Fritz et al. (2009) reported that members of the Mafa tribe, who live in the northern parts of Cameroon (without electricity or any access to Western media like radio and TV), are able to recognize emotional expressions of happiness, sadness or fear above chance level in Western music. In addition to *expressing* emotion, music has often been shown to have an impact on several response components of *induced* emotion (Grewe et al., 2007; Salimpoor et al., 2011; Egermann et al., 2013), and emotional responses have been reported in Western cultures as one of the primary motivations to engage in musical activities (Schäfer et al., 2013). In the present study, we explored whether universal effects of music could also be found for psychophysiological measures of emotion induction to extend existing findings on universal emotional recognition.

Theories of emotion induction through music suggest that responses are generated within a framework of several mechanisms that independently generate emotional responses to music (Juslin and Västfjäll, 2008; Juslin et al., 2010): cognitive appraisal of music and the listening situation, visual imagery induced through sound, evaluative conditioning from pairing music to another emotion-inducing stimulus, emotional episodic memory associated with the music, violation of musical expectations, emotional contagion through emotional expressions in the music, entrainment of bodily rhythms to recurring periodicities in the music, and brainstem reflexes to low-level acoustic characteristics of the music. Although some of these mechanisms still require empirical testing, the validity of others has been confirmed by previous research. For example, Egermann et al. (2013) report that unexpected musical events, identified through simulations of auditory statistical learning, induced reactions in the emotional response components of physiological arousal and subjective feeling (Scherer, 2005). This was indicated by increases in skin conductance, decreases in heart rate, and increases (decreases) in subjective arousal (valence) ratings. Induction mechanisms that are based on memory are thought to be highly influenced by individual and cultural learning (such as evaluative conditioning, episodic memory, or musical expectancy). However, other mechanisms of emotion induction have been described as being based on culture-independent universal response patterns (Juslin and Västfjäll, 2008). Potential candidates for such universal mechanisms include emotional contagion (see also Egermann and McAdams, 2013), rhythmic entrainment, and brainstem reflexes. The universality of brainstem reflexes was partially shown by Fritz et al. (2009), who reported that spectral manipulations of music recordings that increased sensory dissonance universally led to decreased ratings of pleasantness in both Mafa and Western groups. However, the cultural independence of emotional contagion and rhythmic entrainment has yet to be proven. All three culture-independent mechanisms are based on several low-level acoustical features of music such as changes in loudness, tempo, timbre, and pitch, which have been shown to be significantly related to psychophysiological indicators of emotional response in Western listeners (Gomez and Danuser, 2007). Furthermore, previous research indicates that these effects might generalize to other cultures, as similar emotion-specific autonomic nervous system activity occurs in participants with different cultural backgrounds (Levenson, 1992; Breugelmans et al., 2005; Soto et al., 2005).

We ran two identical experiments within two cultural settings: one during a field study of the Mbenzélé Pygmies in the northern Congo and another one within a Western group of Canadian music listeners. The Pygmies live isolated in small hunter-gatherer communities in the rainforest. Because of the lack of electricity, they do not have access to radio, television, or electricity, and thus are completely unfamiliar with Western music.

We measured activity in the three response components of emotion (feeling, physiological activation, and expression, Scherer, 2005) when presenting Mbenzelé and Western music to both cultural groups. After exploring their subjective emotional responses to the music, we tested for response similarity by comparing physiological responses to Western and Pygmy stimuli, which were rated with respect to their relative arousal and valence by the respective culturally familiar participant group. Furthermore, we tested whether responses across groups would be similarly related to low-level acoustic features of the music, which could suggest the operation of culture-independent response mechanisms.

## Methods

### Participants

Forty Mbenzélé Pygmies (22 males, *M* = 35 years, *SD* = 14) and 40 Canadians (22 males, *M* = 22 years, *SD* = 6) participated in the two experiments. We recruited as many Mbenzélé Pygmy participants as there were villagers available that were interested in participating. Subsequently, the number of Canadian participants was matched to that of Pygmy participants. The Pygmies regularly engage in local musical activity for ceremonial purposes, and everyone participates actively in music making. Therefore, Canadian participants were recruited as amateur or professional musicians who were also regularly engaging in musical activity. No participants reported any hearing impairments. Both participant groups received financial compensation for participation. It is important to note that the Pygmies have no contact with Western music and minimal contact with popular music from Zaïre when traveling to nearby towns to trade. They do have also contact with the music of the Bantu people in the region. Their music is similar in some ways but the Pygmies make a point of honor in distinguishing their music from Bantu music. In particular, the Bantus do not produce the complex polyphonies unique to the Pygmy music in all of Africa.

### Musical stimuli

The stimulus materials consisted of 19 musical excerpts taken from both Western and Congolese Pygmy repertoires plus one test Western excerpt (see Table S1). Eight Western orchestral musical excerpts were taken from a previous study that grouped pieces according to similar emotional meaning (Bigand et al., 2005). We selected two stimuli per emotion quadrant from the two-dimensional emotion model (Russell, 1980), which represents emotion along the dimensions of valence (negative to positive) and arousal (calm to excited). Additionally, three excerpts were experimenter-selected from popular Western films to express three basic emotions: happiness, fear, and sadness. The Pygmy music was recorded in the field. It is well known for its polyphonic complexity, often sung on pentatonic scales (Shebasta, 1952; Turnbull, 1961; Arom, 1991). A Pygmy music expert selected eight typical vocal pieces that are usually performed in different ceremonial contexts that often had emotional connotations, such as being sung in order to calm down anger or fear or to alleviate bereavement. Stimulus duration ranged from 30 s to 2 min, and all were presented in random order.

### Measurements

An iPod Touch (Apple Inc., Cupertino, CA) was used to continuously rate subjective feeling during stimulus presentation on a two-dimensional emotion space. The horizontal axis was a representation of valence, the relative negative or positive nature of an emotion, and the vertical axis was a representation of emotional arousal, a spectrum from calm to excited. The heuristic value of the two-dimensional emotion space was previously confirmed in numerous other studies measuring emotion in music (Schubert, 1999; Nagel et al., 2007; Egermann et al., 2009, 2013). Participants were instructed to start rating for every excerpt in the center of the emotion space and then to move their finger according to their current emotional state. To denote the emotional qualities of the emotion space, illustrated faces depicting a negative (upside-down smile) and positive (smile) face were placed on the extreme ends of the horizontal axis outside the iPod. Aroused (open eyes) and calm (closed eyes) faces were placed on the extreme ends of the vertical axis. Participants were instructed to rate their own felt emotions caused by the music, and not to rate what they thought they recognized as an emotional expression in the music. At the beginning of the field trip in the Congo, all Pygmies who were interested in participating were invited to an extensive collective presentation describing the experiments, and instructions were given. Additionally, all instructions were repeated right before each experiment. Physiological measurements were recorded through ProComp Infiniti units (Thought Technology Ltd, Montreal, QC). Respiration was measured using a respiration belt attached around the chest. Blood volume pulse (BVP) was measured using a photoplethysmograph on the palmar side of the distal phalange of the middle finger of the non-dominant hand. Electrodermal activity was measured using electrodes on the distal phalanges of the index and ring fingers of the non-dominant hand. Expressive muscle activations were measured using two electromyography electrodes (MyoScan-Pro surface sensors) placed on the corrugator supercilii (frowning) and zygomaticus major (smiling) muscles (Cacioppo et al., 1986).

### Procedure

The research reported in this manuscript was carried out according to six the principles expressed in the Declaration of Helsinki and the Research Ethics Board of McGill University has reviewed and approved this study (certificate #156-0107).

In order to create identical testing settings, the procedure was kept constant between experiments with both subpopulations. To ensure comfort for the Pygmies in the unfamiliar testing situation, all participants had to be tested in pairs of close friends or family members. The two participants were seated on opposite sides of a table facing each other, with the experimenter on the side in between them. Experiments in Canada were conducted in English. For the Congolese testing session, an interpreter was seated opposite the experimenter (translating between the Mbenzélé language of the Pygmies and French, the first language of the experimenter). There was a small barrier between participants preventing them from seeing each other's ratings. After biosensors were attached, baseline physiological activity during 2 min of silence was recorded, followed by the practice trial. The stimuli were presented through a pair of MDR-NC7 headphones (Sony Corporation, Tokyo, Japan) at a comfortable listening level for one third of participants of both groups. For the rest of the participants, stimuli were presented through built-in Macbook Pro speakers at maximal volume, because of technical difficulties encountered during testing in the field in the Congo rainforest, where a pair of headphones broke after the fourteenth participant. After every stimulus, participants in both experiments were asked, if they knew the music previously presented to them. Here, all Canadian participants responded that they did not know or had not heard the Pygmy excerpts before, whereas all Pygmy participants indicated that they did not know or had not heard the Western excerpts before.

### Data analysis

After removal of visually erroneous datasets due to movement-related sensor displacement (per excerpt and participant), we extracted averaged response scores per music excerpt, signal and participant. First, BVP (low pass 2 Hz), respiration activity (low pass 1 Hz), and skin conductance (low pass 0.3 Hz) signals were filtered using Matlab (Version 8.14.0.604, The Mathworks Inc., Natick, MA) in order to remove extraneous information using a linear-phase filter based on the convolution of a 4th-order Butterworth filter impulse response (also convolved with itself in reverse time to avoid phase shifting). The MyoScan-Pro EMG sensors automatically converted their signal to a root mean square (RMS) signal (after an internal analog rectification), which was therefore not filtered any further (capturing EMG activity at frequencies up to 500 Hz). As there were several errors in the EMG corrugator supercilii recordings in the Congolese sessions, we are not presenting any results from these measurements. We performed linear detrending on the skin conductance signal in order to remove any negative trends over time with breakpoints every 60 s. [These trends are caused by an accumulation of charge over time between the skin and sensor (Salimpoor et al., 2009)]. Skin conductance response events were subsequently computationally identified at onsets of skin conductance increases of any size. In order to remove any between participant differences in physiological baseline activity, we subtracted the corresponding baseline response score for every participant from all of her/his stimulus response scores. Furthermore, we calculated the mean arousal and valence rating per participant and excerpt excluding a 7-s orientation phase after stimulus onset, which was identified after visual inspection of continuous rating data (Bachorik et al., 2009; Schubert, 2012).

This procedure resulted in seven different mean response scores per participant and music excerpt: heart rate, skin conductance level (SCL), number of skin conductance responses per minute (SCR), respiration rate, EMG zygomaticus activation, and arousal and valence ratings. However, before using these resulting response score vectors in subsequent analyses, they were z-standardized across excerpts and participants.

For testing of significance on response scores, we employed a hierarchical linear modeling approach (West et al., 2006) using the MIXED procedure in SPSS Statistics (IBM, Version 21). Estimation of parameters was based on restricted maximum likelihood. Beside fixed effects coefficients, the models included an intercept and a first-order autoregressive residual covariance structure modeling carryover effects from previous stimulus trials (AR1).

## Results

### Effects of musical excerpts on subjective feeling

**Figure 1:**
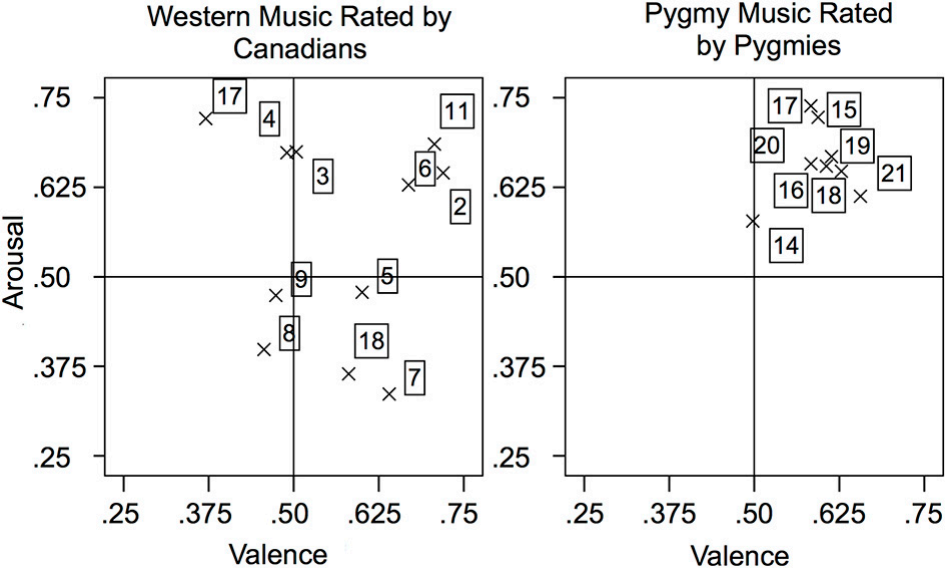
**Mean arousal and valence ratings separated by music excerpt and participant group**. High valence was defined as positive, low valence as negative.

Figure 1 presents the mean arousal and valence rating per excerpt, separated by stimulus cultural origin and participant group in the two-dimensional emotion space. The Western music rated by Canadian participants covered all four quadrants: pieces were rated with both high and low subjective arousal, and some induced a positive affect whereas others induced a negative affect. In contrast, Pygmies rated most Pygmy music excerpts on average as both positive and arousing (except excerpt 14). However, within this quadrant, pieces can still be categorized relatively as more or less arousing than others, and as more or less positive than others.

### Effects of western musical excerpts

We subsequently tested whether subjective ratings of the Western stimuli by the Canadian participant group were similarly related to the subjective and physiological response signals recorded in both groups. Therefore, we estimated two hierarchical linear models for every response score, one for each of the Canadian and Pygmy participant groups (Equation 1):

(1) $$\text{response score}={\text{b}}_{0}+{\text{b}}_{1}\times \text{A}+{\text{b}}_{2}\times \text{V}+{\text{b}}_{3}\times \text{A}\times \text{V}$$

**Figure 2:**
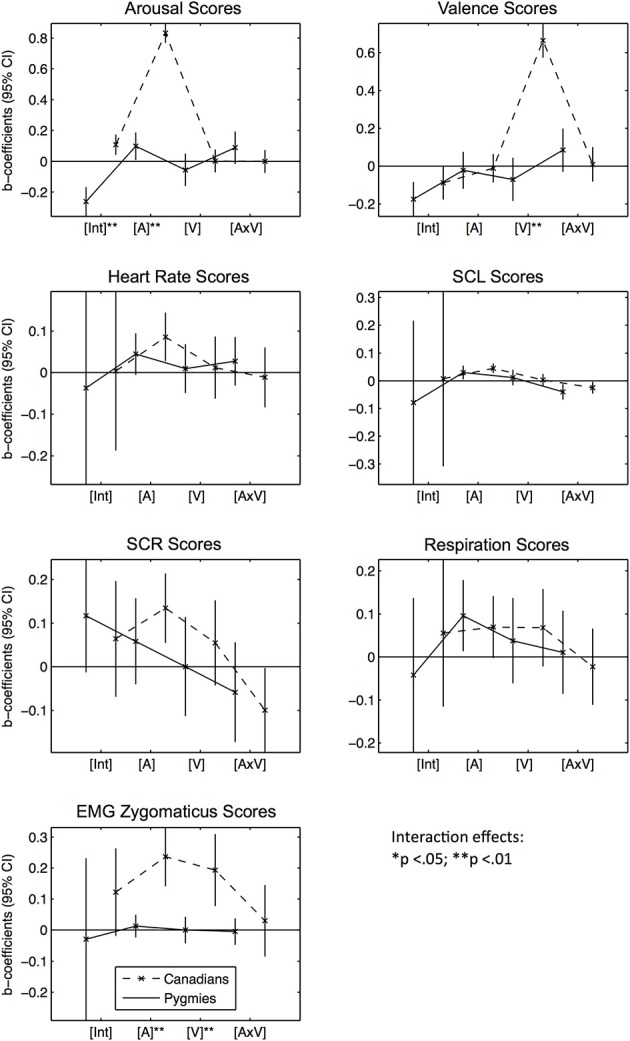
**Error bar graphs of fixed effect coefficients estimated for Western music excerpts' arousal and valence ratings separated by participant group and response score types**. CI, confidence interval; [Int], Intercept; [A], Mean arousal rating of excerpt by Canadian participant group; [V], Mean valence rating of excerpt by Canadian participant group; [V × A], Interaction effect between [A] and [V]. The solid line represents effect estimates for Pygmies, the dashed line represents effects for Canadians. Asterisks indicate significance of fixed effect differences between Pygmy and Canadian groups: ^*^p < 0.05, ^**^p < 0.01 (two sided *t*-tests).

As fixed effects (b_0_ to b_3_), we added an intercept [Int], the Western groups' mean arousal [A] and valence [V] ratings (per excerpt), and an interaction between the two, modeling an additional effect of the co-occurrence of positive and arousing feelings [A × V]. Subsequently, we also tested if the effect coefficients estimated for the two participant groups significantly differed from each other through estimating a third model with interaction effects between the fixed effects [A,V,A × V] on the one side and being in the Pygmy participant group (dummy variable with being Canadian as a reference group) on the other. In these models, we also added being in the Pygmy participant group as a fixed effect, modeling significant differences between response score group means that were independent of any music excerpt. Figure 2 visually presents the estimated fixed-effects coefficients as error-bar graphs (see Table S2 for statistical details). The effects displayed are the two Intercepts for both groups [Int], followed by the arousal [A], valence [V], and interaction [A × V] effects. A significant effect (greater or smaller than zero) is indicated by the fact that the confidence interval does not overlap with the zero line. Significant differences of fixed effects between participant groups are indicated with asterisks.

*Subjective Arousal Scores*: In general, Canadians rated the Western music as more arousing than the Pygmy group, indicated by a significant difference between group means [Int]. However, Canadian arousal ratings [A] are a significant predictor of Pygmy arousal ratings: for excerpts that Canadians rate as more arousing than other excerpts, Pygmies also rated them as arousing. No other effects are significant. *Subjective Valence Scores:* Only intercepts [Int] in both groups are significantly lower than zero. *Heart Rate Scores:* Music rated as more arousing by Canadians elicited increased heart rates in both groups [A], although for Pygmies this was only a non-significant trend (*p* < 0.10). *SCL Scores:* Both groups responded similarly to increases in Arousal [A]: higher arousal ratings by Canadians for a given piece were related to higher skin conductance levels in both groups. When the Western music was rated as positive and arousing at the same time [A × V], both groups surprisingly responded with decreased skin conductance. *SCR Scores:* Only Canadian participants responded with an increased number of skin conductance responses when they rated the music as arousing [A]. *Respiration Scores:* Both groups responded with increased respiration rates when the Western music was arousing compared to calming [A]. *EMG Zygomaticus Scores:* Only Canadians responded with increased zygomaticus activation when the Western music was arousing [A] and positive [V].

Summarized together, these results indicate that there was a positive correlation between subjective arousal ratings of Canadians and Pygmies that was accompanied in both groups by increases in physiological arousal (heart rate, skin conductance, and respiration rate). There were no similarities between the two participant groups in responses to stimuli that were rated as having different degrees of valence.

### Effects of pygmy music excerpts

**Figure 3:**
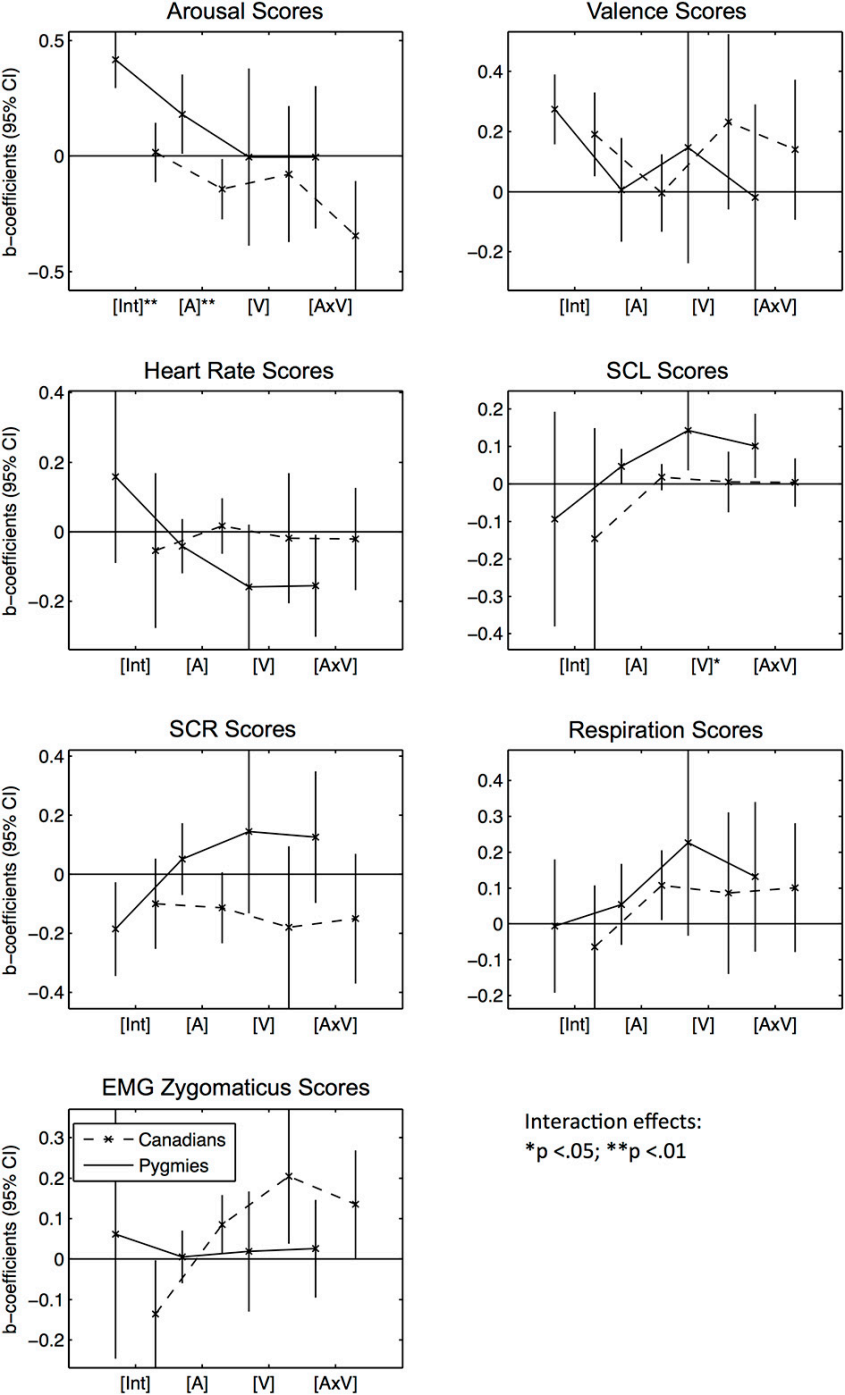
**Error bar graphs of fixed effect coefficients estimated for Pygmy music excerpts' arousal and valence ratings separated by participant group and response score types**. CI, confidence interval; [Int], Intercept; [A], Mean arousal rating of excerpt by Pygmy participant group; [V], Mean valence rating of excerpt by Pygmy participant group; [V × A], Interaction effect between [A] and [V]. The solid line represents effect estimates for Pygmies, the dashed line represents effects for Canadians. Asterisks indicate significance of fixed effect differences between Pygmy and Canadian groups: ^*^p < 0.05, ^**^p < 0.01 (two sided *t*-tests).

We subsequently tested for effects of Pygmy music excerpts using model configurations that were identical to the Western excerpt analyses, except that now Pygmies' subjective excerpt ratings were used as fixed effects (Figure 3 and Table S3).

*Subjective Arousal Scores:* In general, Pygmies rated their own music as significantly more arousing than did Canadians [Int]. Arousing Pygmy excerpts (vs. less arousing excerpts as rated by Pygmies, [A]) were negatively related to arousal ratings in Canadians, as were excerpts that were rated as arousing and positive [A × V]. *Subjective Valence Scores:* No effect was significant in the valence model, except the two intercepts [Int], which were both positive. *Heart Rate Scores:* Only Pygmies responded with decreased heart rates when they listened to excerpts that were arousing and positive to them [A × V]. *SCL Scores:* Only Pygmies responded with increased skin conductance when they rated some excerpts as more arousing and positive than other excerpts [A, V, A × V]. *SCR Scores:* No significant effect could be observed here. *Respiration Scores:* Canadians responded with increased breathing rates to music the Pygmies rated as more arousing. *EMG Zygomaticus Scores:* Although Pygmies didn't show any significant effects here, Canadian participants responded with increased activation for music rated by Pygmies as arousing and positive [A, V, A × V].

Summarized together, the responses to Pygmy excerpts indicate that participant groups responded very differently to the music. Changes in arousal and valence [A, V] were only associated with changes in heart rate and skin conductance for Pygmies. However, Canadians responded with increases in respiration rate to increases in excerpt arousal [A]. Interestingly, Canadians also responded with increases in zygomaticus activity to increases in excerpt arousal [A] and valence [V, A × V]. Thus, there were little similarities between the two participant groups in responses to the emotional qualities of Pygmy music.

### Acoustical descriptor effects of music excerpts

Given that we observed some similarities between the two participant groups in responding to the musical excerpts, we subsequently tested whether these similarities could be explained by similar responses to underlying low-level acoustical features of the stimuli. We extracted six acoustical descriptors averaged across each excerpt using the MIR Toolbox v1.4.1 (Lartillot et al., 2008): This included roughness based on the Sethares-method, the centroid of the spectral distribution, mode as the relative fit between a major or minor chromagram (which may be difficult to extract from the Pygmy music), dominant pitch based on an autocorrelation function of the audio waveform, and event density as the number of note onsets per seconds. Tempo (in beats per minute, BPM) was measured by tapping the dominant beat oft the excerpt into an web-based BPM-Tracker. All descriptors were subsequently entered into a Principle Component Analysis (Table 1).

**Table 1:** **Component Loadings from Principal Component Analyses of Acoustical Descriptors of Music Excerpts (*n* = 19)**.

	**Principal Component**					
**Acoustical Descriptor^1^**	**1**	**2**	**3**	**4**	**5**	**6**
RMS Energy^1^	0.97	−0.05	−0.03	0.06	0.07	0.19
Roughness^1^	0.91	−0.02	0.08	−0.02	0.34	0.17
Spectral Centroid^1^	−0.07	0.94	−0.05	0.15	0.21	0.19
Mode^1^	0.03	−0.04	0.99	−0.16	−0.02	0.00
Pitch^1^	0.03	0.14	−0.16	0.97	0.06	−0.01
Event Density^1^	0.31	0.25	−0.03	0.08	0.90	0.16
Beats Per Minute	0.30	0.21	0.00	−0.01	0.16	0.92

Rotation Method: Varimax with Kaiser Normalization.

1 Mean across excerpt.

**Figure 4:**
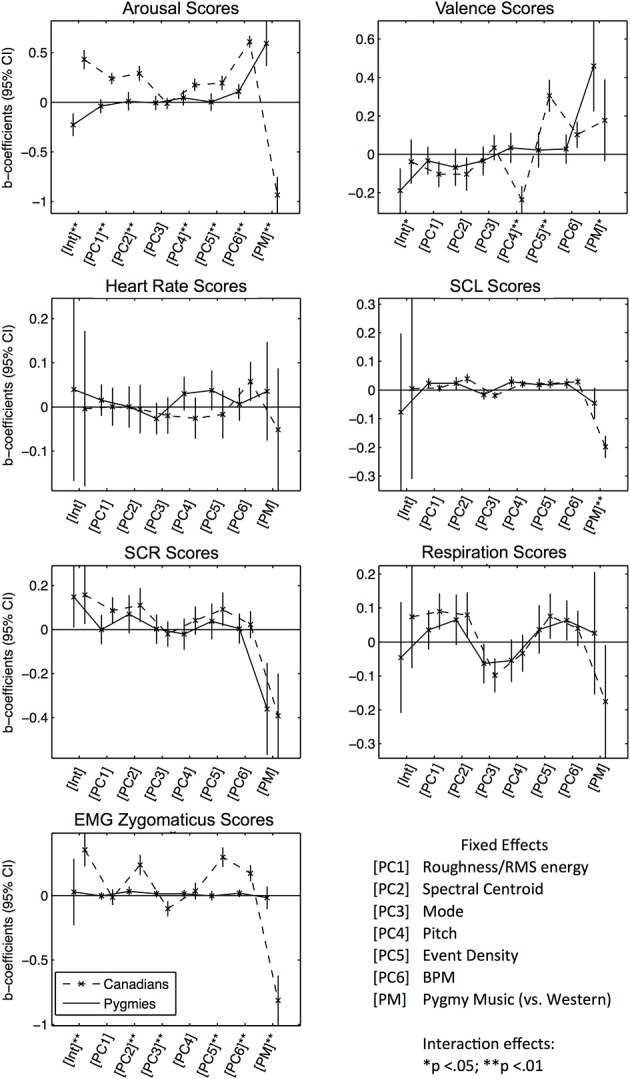
**Error bar graphs of fixed effect coefficients estimated for significant effects of the Acoustical PCs, separated by participant group and response score types**. The solid line represents effect estimates for Pygmies, the dashed line represents effects for Canadians. Asterisks indicate significance of fixed effect differences between Pygmy and Canadian groups: ^*^p < 0.05, ^**^p < 0.01 (two sided *t*-tests).

The resulting six principle components [PC 1–6] were then used as independent predictors in subsequent linear modeling analyses. We also added an additional dummy variable that coded whether the excerpt was from the Pygmy music repertoire or not (with Western music as reference group) [PM]. This was done to account for any other uncontrolled music features that differed between the Western and Pygmy music. Similar to previous analyses, we also tested for significant group differences in responses to acoustical principal components and PM by estimating a third model for every response score with interaction effects between fixed effects and being in the Pymgy group. Figure 4 presents the estimated fixed-effects coefficients (separated by response scores), significant group differences are indicated through asterisks (see Table S4 for statistical details).

*Subjective Arousal Scores*: The Canadian group responded with increased arousal ratings to increases in all PCs, except for mode [PC 3] and PM. The Pygmy group responded only to increases in tempo [PC 6]. Although Canadian participants were more responsive to tempo (indicated by the significantly increased effect size), for both participant groups a positive effect was estimated. This could explain why Pygmies reported increased arousal to Western music that Canadians also rated as arousing. *Subjective Valence Scores:* Although Canadian participants responded to increases in roughness/RMS energy, spectral centroid, and pitch [PCs 1, 2, and 4] with decreased valence and to increases in event density [PC 5] with increased valence, Pygmies did not respond to any acoustical descriptor. They consistently rated their own music as significantly more positive than did Canadians. *Heart Rate Scores:* Only Canadians responded to increased tempo [PC 6] with increased heart rate. *SCL Scores:* Both groups responded with increased skin conductance when spectral centroid, pitch, and tempo increased [PC 2, 4, 6]. Only Pygmies responded with increased SCL when RMS energy and roughness [PC 1] increased, and only Canadians responded to increases in mode (SCL decrease), event density (SCL increase) and the Pygmy music (SCL decrease) [PCs 3, 5, PM]. *SCR Scores:* Only Canadians responded to increased roughness/RMS energy, spectral centroid, and event density [PCs 1, 2, 5] with increases in SCR. Both groups responded with decreased SCR during listening to the Pygmy music. *Respiration Scores:* Canadians responded with increased respiration rates when roughness/RMS energy, spectral centroid, and event density [PCs 1, 2, 5] increased. Note that there are no significant differences for any acoustical PC effect coefficients between the groups for SCL, SCR, and Respiration Scores. The shape of the error graph line is very similar in both groups. However, some effect coefficients are only significant in the Canadian participant group indicating that for Pygmies estimated effects were too small to become significant, potentially due to general increased physiological measurement error in the rain forest field setting. *EMG Zygomaticus Scores:* Only Canadians responded with increased zygomaticus activation to increases spectral centroid, event density, and tempo [PC 2, 5, 6] and only Canadians responded with decreased activation when mode [PC 3] increased (more major) or when listening to Pygmy music [PM].

## Discussion

We tested for effects of group membership (i.e., cultural familiarity), music excerpt qualities (indexed by subjective arousal and valence ratings of excerpts from within cultures), and underlying low-level acoustical descriptors on emotional response to music. The results demonstrate both similarities and differences in participants' responses to different music excerpts, which are likely mediated through different response mechanisms.

The following differences between both groups occurred: Except for the effects of Pygmy music in general, effects in models estimated for SCR scores were never significant in the Pygmy participant group, indicating that this score did not reflect their sympathetic arousal very well. Furthermore, although there were many significant effects on zygomaticus recordings in Canadians, in Pygmies they were not significantly influenced by any independent variable, indicating a) that they did not respond with expressive facial reactions in general (maybe because of increased seriousness due to the unfamiliar experimental setting) or b) that also for this facial muscle, measurement errors occurred in the field setting.

Canadians rated Western music as more subjectively arousing than did Pygmies, whereas Pygmies rated their music as more arousing than Canadians (also illustrated by corresponding effects in SCL scores). This could reflect a general increase in attentional focus for music that is culturally meaningful and familiar. Subjective valence ratings were also different between groups (e.g., Pygmies rated their own music as more positive than Canadians and did not respond with changes in valence to any acoustical descriptor of the music). Only Pygmies responded with increases in physiological arousal (SCL) when they rated their own music as more arousing and positive. These findings suggest that such response differences were mediated by mechanisms based on cultural learning (cognitive appraisal, episodic memory, or musical expectancy).

There were several similarities in both groups' responses to the different musical stimuli. When Canadians rated Western music as subjectively arousing, both groups responded with increased physiological arousal (heart rate, skin conductance, and respiration). Additionally, in Pygmies, subjective arousal ratings increased for arousing Western music. Taken together, these similarities observed in emotional responding could be explained by universal reaction patterns to several low-level features. Both groups responded with increased subjective and physiological arousal (SCL) when the music had a higher tempo. Furthermore, increases in spectral centroid and pitch lead to increased skin conductance for both participant groups. Although respiration might have influenced skin conductance (Rittweger et al., 1997), these relationships might reflect increases in sympathetic arousal that could be caused by the brainstem reflex, rhythmic entrainment, or emotional contagion mechanisms, which are thought to be rather independent from cultural learning (Juslin and Västfjäll, 2008).

Increases in tempo may have led to synchronization of internal body rhythms (Juslin et al., 2010), leading to increased arousal (rhythmic entrainment). However, tempo represents a feature that has been shown to co-vary with emotional expression in music (Juslin and Laukka, 2003), which might lead to emotional contagion, a mechanism that is also thought to be weakly influenced by cultural learning. Thus, the universal responses to tempo could also be explained by internal mimicking of the emotional expressions heard in the music, as emotion recognition has been previously been shown to be based to some degree on universal features (Fritz et al., 2009; Laukka et al., 2013).

Increases in pitch and spectral centroid (timbral brightness) are also associated with emotion expression and could thus be associated with arousal because of emotional contagion. However, they might also have a direct influence on arousal because of the brainstem reflexes that react to urgent and important events as described by Juslin and Västfjäll (2008). Excerpt 17 in particular, from the soundtrack of the film “Psycho,” features unusually high and bright violin sounds that could be influential here.

One might add that a brainstem-reflex-mediated arousal response could also influence emotion recognition, as some theories of emotion recognition suggest that it is based on the self-perception of simulated emotional resonance (Cochrane, 2010). To summarize, both routes of emotion induction previously described remain plausible: (a) brainstem reflex and rhythmic entrainment create physiological arousal that is then integrated into a conscious feeling or (b) expressions are internally mimicked and lead to induced emotion with associated responses (emotional contagion).

Contrary to Fritz et al.'s (2009) findings of universal reaction patterns in pleasantness/valence to changes in sensory dissonance/roughness, the universal response patterns observed in this study were mostly related to emotional arousal. This may of course be due to less extreme variability of roughness in our natural stimuli that were not artificially manipulated. However, it might also be that the low-level emotional processing observed in this study is only influential in creating underlying sympathetically mediated arousal responses that still require conscious interpretation into emotional qualities (Schachter and Singer, 1962). Physiological measures of activation indicate much fewer differences between the participant groups. In only one linear model, the interaction term testing for group differences was significantly different from zero (increases in SCR due to increases in Pygmy music valence rating). This suggests that subjective emotional ratings might have been more subject to cultural influences than physiological responses to the stimuli, a finding that is similar to that of Soto et al. (2005): Chinese American participants reported less extreme emotions than did Mexican Americans, whereas emotional behavior and physiology were less differentiated.

The findings from this study are subject to limitations. Firstly, in order to test for effects of natural stimuli, several of their acoustical descriptors co-vary and their relative effects can only be separated in a limited way. However, even being able to differentiate between acoustical descriptors would not allow us to separate psychological emotion-induction mechanisms from each other (Juslin and Västfjäll, 2008), as many acoustical features contribute as inputs to several mechanisms at the same time. Thus, many conclusions on the operation of response mechanisms have to remain speculative. Differences between measurements from the two groups might stem from different responding, but also from other co-varying features of the two measurement situations. Even though we tried to keep as many variables as constant as possible, Pygmies were still very unfamiliar with the experimental procedure, as they were not used to using rating interfaces or listening to recorded music. Additionally, emotional self-reports might be biased by a different understanding of emotional qualities (Matsumoto and Yoo Hee, 2007). However, the experimenter assured through several lengthy interviews that a similar understanding of basic emotional qualities was present in the Pygmy population. When asked if they had ever heard the Western music, they all answered with a categorical “no.” They always expressed appreciation for the Western music (“It's good music!”) but when asked what it expresses, they answered “I don't know, it's your music, you should know.”

The present findings indicate that although valence responses were often different between the two participant groups, music-induced arousal responses appeared to be based on rather universal, culturally independent response mechanisms. These may be based on low-level acoustical characteristics of music like tempo, pitch, or timbre. There were more similarities in participants' responses in Western than in Pygmy music, indicating that Western music may make stronger use of these characteristics than Pygmy music. Five out of the six acoustical principal components extracted from stimuli showed a higher variance in the selected Western music compared to the Pygmy music. It is possible that the communication of emotion in Pygmy music is instead based more on symbolic and associative meaning, mediated through learning. Summarized together, these findings may help to understand the ubiquitously experienced emotional responses to music that can be sometimes very personal and individual, but at other times to some extent also universal and collective.

### Conflict of interest statement

The authors declare that the research was conducted in the absence of any commercial or financial relationships that could be construed as a potential conflict of interest.

## References

[B1] Arom S. (1991). African Polyphony and Polyrhythm: Musical Structure and Methodology. Cambridge, MA: Cambridge University Press.

[B2] Bachorik J. P. Bangert M. Loui P. Larke K. Berger J. Rowe R. (2009). Emotion in motion: investigating the time-course of emotional judgments of musical stimuli. Music Percept. 26, 355–364 10.1525/mp.2009.26.4.355

[B3] Balkwill L.-L. Thompson W. F. (1999). A cross-cultural investigation of the perception of emotion in music: psychological and cultural cues. Music Percept. 17, 43–64 10.2307/40285811

[B4] Balkwill L.-L. Thompson W. F. Matsunaga R. (2004). Recognition of emotion in Japanese, Western, and Hindustani music by Japanese listeners. Jpn. Psychol. Res. 46, 337–349. 10.1111/j.1468-5584.2004.00265.x23398579

[B5] Bigand E. Vieillard S. Madurell F. Marozeau J. Dacquet A. (2005). Multidimensional scaling of emotional responses to music: the effect of musical expertise and of the duration of the excerpts. Cogn. Emot. 19, 1113–1139 10.1080/02699930500204250

[B6] Breugelmans S. M. Poortinga Y. H. Ambadar Z. Setiadi B. Vaca J. B. Widiyanto P.. (2005). Body sensations associated with emotions in Rarámuri Indians, rural Javanese, and three student samples. Emotion 5, 166–174. 10.1037/1528-3542.5.2.166 15982082

[B7] Cacioppo J. T. Petty R. E. Losch M. E. Kim H. S. (1986). Electromyographic activity over facial muscle regions can differentiate the valence and intensity of affective reactions. J. Pers. Soc. Psychol. 50, 260–268. 10.1037/0022-3514.50.2.260 3701577

[B8] Cochrane T. (2010). Music, emotions and the influence of the cognitive sciences. Philos. Compass 11, 978–988 10.1111/j.1747-9991.2010.00337.x

[B9] Egermann H. McAdams S. (2013). Empathy and emotional contagion as a link between recognized and felt emotions in music listening. Music Percept. 31, 137–153 10.1525/mp.2013.31.2.139

[B10] Egermann H. Nagel F. Altenmüller E. Kopiez R. (2009). Continuous Measurement of musically-induced emotion: a web experiment. Int. J. Internet Sci. 4, 4–20.

[B11] Egermann H. Pearce M. T. Wiggins G. A. McAdams S. (2013). Probabilistic models of expectation violation predict psychophysiological emotional responses to live concert music. Cogn. Affect. Behav. Neurosci. 13, 533–553. 10.3758/s13415-013-0161-y 23605956

[B12] Eibl-Eibesfeldt I. (1979). Human ethology: concepts and implications for the sciences of man. Behav. Brain Sci. 2, 1–57 10.1017/S0140525X00060416

[B13] Fritz T. Jentschke S. Gosselin N. Sammler D. Peretz I. Turner R.. (2009). Universal recognition of three basic emotions in music. Curr. Biol. 19, 573–576. 10.1016/j.cub.2009.02.058 19303300

[B14] Gomez P. Danuser B. (2007). Relationships between musical structure and psychophysiological measures of emotion. Emotion 7, 377–387. 10.1037/1528-3542.7.2.377 17516815

[B15] Grewe O. Nagel F. Kopiez R. Altenmüller E. (2007). Emotions over time: synchronicity and development of subjective, physiological, and facial affective reactions to music. Emotion 7, 774–788. 10.1037/1528-3542.7.4.774 18039047

[B16] Harwood D. (1976). Universals in music: a perspective from cognitive psychology. Ethnomusicology 20, 521–533 10.2307/851047

[B17] Huron D. (2008). Lost in music. Nature 453, 456–457. 10.1038/453456a 18497806

[B18] Juslin P. N. Laukka P. (2003). Communication of emotions in vocal expression and music performance: different channels, same code? Psychol. Bull. 129, 770–814. 10.1037/0033-2909.129.5.770 12956543

[B19] Juslin P. N. Liljeström S. Västfjäll D. Lundqvist L.-O. (2010). How does music evoke emotions? Exploring the underlying mechanisms, in Handbook of Music and Emotion: Theory, Research, Applications, eds Juslin P. N. Sloboda J. A. (Oxford: Oxford University Press), 605–643.

[B20] Juslin P. N. Västfjäll D. (2008). Emotional responses to music: the need to consider underlying mechanisms. Behav. Brain Sci. 31, 559–75; discussion 575–621. 10.1017/S0140525X08005293 18826699

[B21] Lartillot O. Toiviainen P. Eerola T. (2008). A matlab toolbox for music information retrieval, in Data Analysis, Machine Learning and Applications, Studies in Classification, Data Analysis, and Knowledge Organization, eds Preisach C. Burkhardt H. Schmidt-Thieme L. Decker R. (Springer-Verlag).

[B22] Laukka P. Eerola T. Thingujam N. S. Yamasaki T. Beller G. (2013). Universal and culture-specific factors in the recognition and performance of musical affect expressions. Emotion 13, 434–449. 10.1037/a0031388 23398579

[B23] Levenson R. W. (1992). Autonomic nervous system differences among emotions. Psychol. Sci. 3, 23–27 10.1111/j.1467-9280.1992.tb00251.x

[B24] Matsumoto D. Yoo Hee S. (2007). Methodological considerations in the study of emotion across cultures, in Handbook of Emotion Elicitation and Assessment, eds Coan J. A. Allen J. J. (Oxford: Oxford University Press), 332–348.

[B25] Nagel F. Kopiez R. Grewe O. Altenmüller E. (2007). EMuJoy: software for continuous measurement of perceived emotions in music. Behav. Res. Methods 39, 283–290. 10.3758/BF03193159 17695356

[B26] Rittweger J. Lambertz M. Langhorst P. (1997). Influences of mandatory breathing on rhythmical components of electrodermal activity. Clin. Physiol. 17, 609–618. 10.1046/j.1365-2281.1997.00058.x 9413648

[B27] Russell J. A. (1980). A circumplex model of affect. J. Pers. Soc. Psychol. 39, 1161–1178 10.1037/h0077714

[B28] Salimpoor V. N. Benovoy M. Larcher K. Dagher A. Zatorre R. J. (2011). Anatomically distinct dopamine release during anticipation and experience of peak emotion to music. Nat. Neurosci. 14, 257–262. 10.1038/nn.2726 21217764

[B29] Salimpoor V. N. Benovoy M. Longo G. Cooperstock J. R. Zatorre R. J. (2009). The rewarding aspects of music listening are related to degree of emotional arousal. PLoS ONE, 4:e7487. 10.1371/journal.pone.0007487 19834599 PMC2759002

[B30] Schachter S. Singer J. (1962). Cognitive, social, and physiological determinants of emotional state. Psychol. Revi. 69, 379–399. 10.1037/h0046234 14497895

[B31] Schäfer T. Sedlmeier P. Städtler C. Huron D. (2013). The psychological functions of music listening. Front. Psychol. 4:511. 10.3389/fpsyg.2013.00511 23964257 PMC3741536

[B32] Scherer K. R. (2005). What are Emotions? And how can they be measured? Soc. Sci. Inf. 44, 695–729 10.1177/0539018405058216

[B33] Schubert E. (1999). Measuring emotion continuously: validity and reliability of the two-dimensional emotion-space. Aust. J. Psychol. 51, 154–165 10.1080/00049539908255353

[B34] Schubert E. (2012). Reliability issues regarding the beginning, middle and end of continuous emotion ratings to music. Psychol. Music, 41, 350–371 10.1177/0305735611430079

[B35] Shebasta P. (1952). Les Pygmées du Congo Belge. Brussels: Institut Royal Colonial Belge.

[B36] Sievers B. Polansky L. (2013). Music and movement share a dynamic structure that supports universal expressions of emotion. Proc. Natl. Acad. Sci. U.S.A. 110, 70–75. 10.1073/pnas.1209023110 23248314 PMC3538264

[B37] Soto J. A. Levenson R. W. Ebling R. (2005). Cultures of moderation and expression: emotional experience, behavior, and physiology in Chinese Americans and Mexican Americans. Emotion 5, 154–165. 10.1037/1528-3542.5.2.154 15982081

[B38] Turnbull C. M. (1961). The Forest People. New York, NY: Simon and Schuster.

[B39] West B. T. Welch K. B. Galecki A. T. (2006). Linear Mixed Models: a Practical Guide Using Statistical Software. Chapman and Hall/CRC Press.

