# Supplementary material for: Music induces universal emotion-related psychophysiological responses: comparing Canadian listeners to Congolese Pygmies
Source: Front Psychol. 2015 Jan 7;5:1341. doi: 10.3389/fpsyg.2014.01341 (PMC4286616; doi:10.3389/fpsyg.2014.01341)
Supplement: Supplementary file 1 [file Table1.DOCX]

Table S1.

*List of Music Excerpts Used*

| Stimulus Number | Stimulus | Source | Duration |
| --- | --- | --- | --- |
|  | Western Music |  |  |
| *test* | *(D. Shostakovitch. Symphony 15, Adagio.)* | B | 00:37 |
| 2 | F. Mendelssohn, Italian Symphony, First Movement | B | 01:14 |
| 3 | F. Liszt, Tasso Lamento & Triomfo | B | 00:30 |
| 4 | R. Strauss, Tod & Verklaerung | B | 00:30 |
| 5 | J.S. Bach, Violin Sonata 3, Fuga | B | 00:45 |
| 6 | I. Stravinsky, Petrouchka | B | 00:29 |
| 7 | J. Brahms, Violin Concerto, Adagio | B | 00:40 |
| 8 | D. Shostakovitch, Trio 2 for piano, violin and cello | B | 00:50 |
| 9 | R. Wagner, Tristan, Act 3. | B | 00:46 |
| 11 | Music from Cantina – Starwars Soundtrack | E | 00:36 |
| 17 | Psycho, Film Soundtrack | E | 00:30 |
| 18 | Schindler’s List Theme, Soundtrack | E | 00:37 |
|  | Pygmy Music |  |  |
| 10 | Lullaby | P | 01:21 |
| 12 | Music to calm anger | P | 01:13 |
| 13 | Music for Djengui (Mbenzele spirit) | P | 01:08 |
| 14 | Music to entertain | P | 01:23 |
| 15 | Music against fear | P | 01:12 |
| 16 | Music for funerals | P | 01:01 |
| 20 | Music against sadness | P | 01:09 |
| 21 | Music of Yeli for protection of man hunting in forest | P | 01:39 |

*Notes:* Source: B = Bigand et. al (2005), P = Field recordings in pygmy villages, E = experimenter selection/Film music.
