# Supplementary material for: Music induces universal emotion-related psychophysiological responses: comparing Canadian listeners to Congolese Pygmies
Source: Front Psychol. 2015 Jan 7;5:1341. doi: 10.3389/fpsyg.2014.01341 (PMC4286616; doi:10.3389/fpsyg.2014.01341)
Supplement: Supplementary file 2 [file Table2.DOCX]

Table S2.

*Fixed Effect Coefficients (b) Estimated for Western Music Excerpts’ Arousal and Valence Ratings Separated by Participant Group and Response Score Types.*

|  | Pygmies | | | | | Canadians | | | | |
| --- | --- | --- | --- | --- | --- | --- | --- | --- | --- | --- |
| Variable | *b* | *SE* | *df* | *t* | *p* | *b* | *SE* | *df* | *t* | *p* |
| Arousal Scores | | | | | | | | | | |
| [Int] | -0.26 | 0.05 | 151.3 | -5.4 | <.001 | 0.11 | 0.03 | 187.9 | 3.2 | 0.001 |
| [A] | 0.1 | 0.05 | 403.8 | 2.1 | 0.034 | 0.83 | 0.03 | 393.5 | 25.7 | <.001 |
| [V] | -0.06 | 0.05 | 400.1 | -1 | 0.298 | <.01 | 0.04 | 432.1 | 0.1 | 0.954 |
| [A]×[V] | 0.09 | 0.05 | 401.9 | 1.6 | 0.102 | <.01 | 0.04 | 430.1 | <.01 | 0.979 |
| Heart Rate Scores | | | | | | | | | | |
| [Int] | -0.04 | 0.12 | 49.3 | -0.3 | 0.749 | <.01 | 0.1 | 63.6 | <.01 | 0.966 |
| [A] | 0.04 | 0.03 | 294 | 1.7 | 0.082 | 0.09 | 0.03 | 279.2 | 2.9 | 0.004 |
| [V] | 0.01 | 0.03 | 295.9 | 0.3 | 0.755 | 0.01 | 0.04 | 292.6 | 0.3 | 0.753 |
| [A]×[V] | 0.03 | 0.03 | 294.5 | 0.9 | 0.359 | -0.01 | 0.04 | 283.5 | -0.3 | 0.753 |
| EMG Zygomaticus Scores | | | | | | | | | | |
| [Int] | -0.03 | 0.13 | 42.8 | -0.2 | 0.821 | 0.12 | 0.07 | 102.7 | 1.7 | 0.09 |
| [A] | 0.01 | 0.02 | 327 | 0.7 | 0.483 | 0.24 | 0.05 | 293.5 | 4.9 | <.001 |
| [V] | <.01 | 0.02 | 329 | <.01 | 0.995 | 0.19 | 0.06 | 336.9 | 3.3 | 0.001 |
| [A]×[V] | <.01 | 0.02 | 328.6 | -0.2 | 0.833 | 0.03 | 0.06 | 316.3 | 0.5 | 0.609 |
| SCL Scores | | | | | | | | | | |
| [Int] | -0.08 | 0.15 | 38.6 | -0.5 | 0.593 | 0.01 | 0.16 | 38.5 | <.01 | 0.966 |
| [A] | 0.03 | 0.01 | 348.2 | 2.4 | 0.017 | 0.05 | 0.01 | 379.3 | 5.1 | <.001 |
| [V] | 0.01 | 0.01 | 348.9 | 0.8 | 0.428 | <.01 | 0.01 | 380.1 | 0.2 | 0.811 |
| [A]×[V] | -0.04 | 0.01 | 348.6 | -2.8 | 0.005 | -0.02 | 0.01 | 379.8 | -2.3 | 0.022 |
| SCR Scores | | | | | | | | | | |
| [Int] | 0.12 | 0.07 | 115.1 | 1.8 | 0.076 | 0.06 | 0.07 | 106.7 | 1 | 0.34 |
| [A] | 0.06 | 0.05 | 347.5 | 1.2 | 0.243 | 0.13 | 0.04 | 301.7 | 3.3 | 0.001 |
| [V] | <.01 | 0.06 | 334.1 | <.01 | 0.992 | 0.05 | 0.05 | 335.6 | 1.1 | 0.27 |
| [A]×[V] | -0.06 | 0.06 | 327.9 | -1 | 0.317 | -0.1 | 0.05 | 318.3 | -2 | 0.044 |
| Respiration Scores | | | | | | | | | | |
| [Int] | -0.04 | 0.09 | 71.3 | -0.5 | 0.639 | 0.06 | 0.09 | 83.7 | 0.6 | 0.521 |
| [A] | 0.1 | 0.04 | 258.3 | 2.3 | 0.024 | 0.07 | 0.04 | 268.3 | 1.9 | 0.06 |
| [V] | 0.04 | 0.05 | 264.6 | 0.8 | 0.454 | 0.07 | 0.05 | 285.3 | 1.5 | 0.137 |
| [A]×[V] | 0.01 | 0.05 | 250.4 | 0.2 | 0.826 | -0.02 | 0.04 | 277.3 | -0.5 | 0.61 |
| Valence Scores | | | | | | | | | | |
| [Int] | -0.17 | 0.05 | 247.9 | -3.8 | <.001 | -0.09 | 0.04 | 177.7 | -2 | 0.05 |
| [A] | -0.02 | 0.05 | 414.4 | -0.4 | 0.675 | -0.01 | 0.04 | 370.7 | -0.3 | 0.78 |
| [V] | -0.07 | 0.06 | 414.8 | -1.2 | 0.23 | 0.66 | 0.05 | 412 | 14.4 | <.001 |
| [A]×[V] | 0.08 | 0.06 | 412.5 | 1.5 | 0.143 | 0.01 | 0.05 | 404 | 0.2 | 0.833 |

*Note:* [Int] = Intercept; [A] = Mean arousal rating of excerpt by Canadian participant group; [V] = Mean valence rating of excerpt by Canadian participant group; [A×V] = Interaction effect between [A] and [V].
