# Supplementary material for: Music induces universal emotion-related psychophysiological responses: comparing Canadian listeners to Congolese Pygmies
Source: Front Psychol. 2015 Jan 7;5:1341. doi: 10.3389/fpsyg.2014.01341 (PMC4286616; doi:10.3389/fpsyg.2014.01341)
Supplement: Supplementary file 3 [file Table3.DOCX]

Table S3.

*Fixed Effect Coefficients (b) Estimated for Pygmy Music Excerpts’ Arousal and Valence Ratings Separated by Participant Group and Response Score Types.*

|  | Pygmies | | | | | Canadians | | | | |
| --- | --- | --- | --- | --- | --- | --- | --- | --- | --- | --- |
| Variable | *b* | *SE* | *df* | *t* | *p* | *b* | *SE* | *df* | *t* | *p* |
| Arousal Scores | | | | | | | | | | |
| [Int] | 0.42 | 0.06 | 199.7 | 6.8 | <.001 | 0.02 | 0.06 | 121.1 | 0.2 | 0.812 |
| [A] | 0.18 | 0.09 | 299.9 | 2.1 | 0.039 | -0.14 | 0.07 | 195.3 | -2.2 | 0.031 |
| [V] | <.01 | 0.2 | 301.2 | <.01 | 0.98 | -0.08 | 0.15 | 217.4 | -0.5 | 0.6 |
| [A]×[V] | <.01 | 0.16 | 302.3 | <.01 | 0.975 | -0.34 | 0.12 | 212.9 | -2.9 | 0.004 |
| Heart Rate Scores | | | | | | | | | | |
| [Int] | 0.16 | 0.12 | 43.2 | 1.3 | 0.205 | -0.05 | 0.11 | 48.9 | -0.5 | 0.629 |
| [A] | -0.04 | 0.04 | 198.7 | -1 | 0.303 | 0.02 | 0.04 | 198 | 0.4 | 0.679 |
| [V] | -0.16 | 0.09 | 203.3 | -1.7 | 0.083 | -0.02 | 0.09 | 201.9 | -0.2 | 0.844 |
| [A]×[V] | -0.15 | 0.07 | 203.8 | -2.1 | 0.039 | -0.02 | 0.07 | 200.9 | -0.3 | 0.78 |
| EMG Zygomaticus Scores | | | | | | | | | | |
| [Int] | 0.06 | 0.15 | 37.1 | 0.4 | 0.686 | -0.14 | 0.07 | 66.6 | -2 | 0.046 |
| [A] | 0.01 | 0.03 | 217.2 | 0.2 | 0.875 | 0.09 | 0.04 | 209.9 | 2.3 | 0.021 |
| [V] | 0.02 | 0.08 | 220.6 | 0.2 | 0.804 | 0.2 | 0.08 | 218.7 | 2.4 | 0.017 |
| [A]×[V] | 0.03 | 0.06 | 220.5 | 0.4 | 0.674 | 0.14 | 0.07 | 217.2 | 2 | 0.046 |
| SCL Scores | | | | | | | | | | |
| [Int] | -0.09 | 0.14 | 38.3 | -0.7 | 0.51 | -0.15 | 0.15 | 38.2 | -1 | 0.322 |
| [A] | 0.05 | 0.02 | 237 | 2 | 0.047 | 0.02 | 0.02 | 261.1 | 1 | 0.315 |
| [V] | 0.14 | 0.05 | 239 | 2.6 | 0.009 | <.01 | 0.04 | 262 | 0.1 | 0.908 |
| [A]×[V] | 0.1 | 0.04 | 238.7 | 2.3 | 0.021 | <.01 | 0.03 | 261.8 | 0.1 | 0.908 |
| SCR Scores | | | | | | | | | | |
| [Int] | -0.19 | 0.08 | 90.3 | -2.3 | 0.022 | -0.1 | 0.08 | 92 | -1.3 | 0.196 |
| [A] | 0.05 | 0.06 | 187.2 | 0.8 | 0.406 | -0.11 | 0.06 | 196.7 | -1.9 | 0.064 |
| [V] | 0.14 | 0.14 | 196.9 | 1 | 0.305 | -0.18 | 0.14 | 211.8 | -1.3 | 0.196 |
| [A]×[V] | 0.13 | 0.11 | 198 | 1.1 | 0.266 | -0.15 | 0.11 | 209 | -1.4 | 0.178 |
| Respiration Scores | | | | | | | | | | |
| [Int] | -0.01 | 0.09 | 69.4 | -0.1 | 0.947 | -0.06 | 0.09 | 65.2 | -0.7 | 0.458 |
| [A] | 0.05 | 0.06 | 175.6 | 1 | 0.342 | 0.11 | 0.05 | 193.8 | 2.2 | 0.03 |
| [V] | 0.23 | 0.13 | 182.8 | 1.7 | 0.086 | 0.09 | 0.11 | 202.6 | 0.8 | 0.452 |
| [A]×[V] | 0.13 | 0.11 | 183.3 | 1.2 | 0.215 | 0.1 | 0.09 | 200.9 | 1.1 | 0.269 |
| Valence Scores | | | | | | | | | | |
| [Int] | 0.27 | 0.06 | 189.6 | 4.7 | <.001 | 0.19 | 0.07 | 105.1 | 2.7 | 0.008 |
| [A] | 0.01 | 0.09 | 276.4 | 0.1 | 0.942 | <.01 | 0.06 | 191.7 | -0.1 | 0.946 |
| [V] | 0.15 | 0.2 | 281.1 | 0.8 | 0.452 | 0.23 | 0.15 | 211.1 | 1.6 | 0.117 |
| [A]×[V] | -0.02 | 0.16 | 274.3 | -0.1 | 0.904 | 0.14 | 0.12 | 207.4 | 1.2 | 0.238 |

*Note:* [Int] = Intercept; [A] = Mean arousal rating of excerpt by Pygmy participant group; [V] = Mean valence rating of excerpt by Pygmy participant group; [A×V] = Interaction effect between [A] and [V].
