# Supplementary material for: Music induces universal emotion-related psychophysiological responses: comparing Canadian listeners to Congolese Pygmies
Source: Front Psychol. 2015 Jan 7;5:1341. doi: 10.3389/fpsyg.2014.01341 (PMC4286616; doi:10.3389/fpsyg.2014.01341)
Supplement: Supplementary file 4 [file Table4.DOCX]

Table S4.

*Fixed Effect Coefficients (b) Estimated for Effects of Acoustical Principal Components (PC) Separated by Participant Group and Response Score Types.*

|  | Pygmies | | | | | | | | | | | | Canadians | | | | | | | |
| --- | --- | --- | --- | --- | --- | --- | --- | --- | --- | --- | --- | --- | --- | --- | --- | --- | --- | --- | --- | --- |
| Variable | *b* | | *SE* | | | *df* | | | *t* | | | *p* | *b* | *SE* | | *df* | | *t* | *p* | |
| Arousal Scores | | | | | | | | | | | | | | | | | | | | |
| [Int] | -0.23 | | 0.06 | | | 625.3 | | | -3.8 | | | <.001 | 0.43 | 0.05 | | 636 | | 8.7 | <.001 | |
| Roughnes/RMS Energy [PC 1] | -0.04 | | 0.04 | | | 719.4 | | | -1.1 | | | 0.271 | 0.24 | 0.03 | | 731.2 | | 8.3 | <.001 | |
| Spectral Centroid [PC 2] | 0.01 | | 0.05 | | | 720 | | | 0.2 | | | 0.844 | 0.29 | 0.04 | | 727 | | 7.6 | <.001 | |
| Mode [PC 3] | -0.01 | | 0.04 | | | 719.2 | | | -0.2 | | | 0.86 | -0.01 | 0.03 | | 673.8 | | -0.4 | 0.702 | |
| Pitch [PC 4] | 0.04 | | 0.04 | | | 719.5 | | | 1.1 | | | 0.251 | 0.17 | 0.03 | | 731.7 | | 5.6 | <.001 | |
| Event Density [PC 5] | <.01 | | 0.05 | | | 719.8 | | | <.01 | | | 0.962 | 0.19 | 0.04 | | 745.2 | | 5.3 | <.001 | |
| Tempo [PC 6] | 0.11 | | 0.04 | | | 719.1 | | | 2.9 | | | 0.004 | 0.61 | 0.03 | | 721 | | 20.3 | <.001 | |
| Pygmy Music [PM] | 0.59 | | 0.12 | | | 716.8 | | | 5.1 | | | <.001 | -0.93 | 0.09 | | 731.8 | | -10 | <.001 | |
| Heart Rate Scores | | | | | | | | | | | | | | | | | | | | |
| [Int] | 0.04 | | 0.1 | | | 63.8 | | | 0.4 | | | 0.707 | <.01 | 0.09 | | 98 | | <.01 | 0.964 | |
| Roughnes/RMS Energy [PC 1] | 0.02 | | 0.02 | | | 540.1 | | | 0.8 | | | 0.4 | <.01 | 0.02 | | 536.1 | | <.01 | 0.974 | |
| Spectral Centroid [PC 2] | <.01 | | 0.02 | | | 539.1 | | | <.01 | | | 0.993 | <.01 | 0.03 | | 525 | | -0.2 | 0.86 | |
| Mode [PC 3] | -0.03 | | 0.02 | | | 535.8 | | | -1.5 | | | 0.145 | -0.02 | 0.02 | | 523 | | -0.9 | 0.353 | |
| Pitch [PC 4] | 0.03 | | 0.02 | | | 540.2 | | | 1.6 | | | 0.121 | -0.03 | 0.02 | | 532.8 | | -1.1 | 0.281 | |
| Event Density [PC 5] | 0.04 | | 0.02 | | | 538.6 | | | 1.6 | | | 0.104 | -0.02 | 0.03 | | 529.9 | | -0.6 | 0.531 | |
| Tempo [PC 6] | 0.01 | | 0.02 | | | 539.6 | | | 0.3 | | | 0.741 | 0.06 | 0.02 | | 528.8 | | 2.6 | 0.011 | |
| Pygmy Music [PM] | 0.04 | | 0.06 | | | 535.7 | | | 0.6 | | | 0.534 | -0.05 | 0.07 | | 527.5 | | -0.7 | 0.469 | |
| EMG Zygomaticus Scores | | | | | | | | | | | | | | | | | | | | |
| [Int] | 0.03 | | 0.13 | | | 45.7 | | | 0.2 | | | 0.822 | 0.36 | 0.07 | | 348.8 | | 5.4 | <.001 | |
| Roughnes/RMS Energy [PC 1] | <.01 | | 0.01 | | | 589.1 | | | -0.1 | | | 0.91 | -0.01 | 0.03 | | 592.2 | | -0.4 | 0.699 | |
| Spectral Centroid [PC 2] | 0.03 | | 0.02 | | | 590.4 | | | 1.7 | | | 0.085 | 0.24 | 0.04 | | 573 | | 6 | <.001 | |
| Mode [PC 3] | 0.02 | | 0.01 | | | 587.7 | | | 1.1 | | | 0.287 | -0.1 | 0.03 | | 536.6 | | -3.3 | 0.001 | |
| Pitch [PC 4] | 0.01 | | 0.02 | | | 591.3 | | | 1 | | | 0.338 | 0.04 | 0.03 | | 576.9 | | 1.1 | 0.284 | |
| Event Density [PC 5] | <.01 | | 0.02 | | | 589.5 | | | <.01 | | | 0.989 | 0.3 | 0.04 | | 601 | | 7.6 | <.001 | |
| Tempo [PC 6] | 0.02 | | 0.01 | | | 589.1 | | | 1.3 | | | 0.192 | 0.17 | 0.03 | | 571.7 | | 5.5 | <.001 | |
| Pygmy Music [PM] | -0.02 | | 0.05 | | | 588.8 | | | -0.4 | | | 0.722 | -0.81 | 0.1 | | 581.3 | | -8.2 | <.001 | |
| SCL Scores | | | | | | | | | | | | | | | | | | | | |
| [Int] | -0.08 | | 0.14 | | | 41 | | | -0.6 | | | 0.576 | 0.01 | 0.16 | | 38.6 | | <.01 | 0.973 | |
| Roughnes/RMS Energy [PC 1] | 0.02 | | 0.01 | | | 633 | | | 2.7 | | | 0.006 | 0.01 | 0.01 | | 693.3 | | 1.1 | 0.278 | |
| Spectral Centroid [PC 2] | 0.02 | | 0.01 | | | 632.9 | | | 2.1 | | | 0.04 | 0.04 | 0.01 | | 692.6 | | 4.9 | <.001 | |
| Mode [PC 3] | -0.02 | | 0.01 | | | 632.3 | | | -1.8 | | | 0.073 | -0.02 | 0.01 | | 692.8 | | -3.2 | 0.002 | |
| Pitch [PC 4] | 0.03 | | 0.01 | | | 632.8 | | | 3.1 | | | 0.002 | 0.02 | 0.01 | | 692.4 | | 3.2 | 0.001 | |
| Event Density [PC 5] | 0.02 | | 0.01 | | | 632.8 | | | 1.7 | | | 0.082 | 0.02 | 0.01 | | 692.9 | | 2.8 | 0.005 | |
| Tempo [PC 6] | 0.02 | | 0.01 | | | 632.7 | | | 2.5 | | | 0.011 | 0.03 | 0.01 | | 693.1 | | 4.5 | <.001 | |
| Pygmy Music [PM] | -0.05 | | 0.03 | | | 632.4 | | | -1.7 | | | 0.094 | -0.2 | 0.02 | | 692.4 | | -10 | <.001 | |
| Table S4 continued. | |  | | | | | | | | | | |  | | | | | | | |
|  | |  | | | | | | | | | | |  | | | | | | | |
|  | | Pygmies | | | | | | | | | | | Canadians | | | | | | | |
| Variable | | *b* | | *SE* | | | *df* | | *t* | | *p* | | *b* | | *SE* | *df* | *t* | | | *p* |
| SCR Scores | | | | | | | | | | | | | | | | | | | | |
| [Int] | | 0.15 | | 0.07 | 360.2 | | | | 2.1 | 0.036 | | | 0.16 | | 0.07 | 348.8 | 2.3 | | | 0.02 |
| Roughnes/RMS Energy [PC 1] | | <.01 | | 0.03 | 580.4 | | | | <.01 | 0.979 | | | 0.09 | | 0.03 | 603.8 | 2.9 | | | 0.004 |
| Spectral Centroid [PC 2] | | 0.07 | | 0.04 | 570.5 | | | | 1.6 | 0.112 | | | 0.11 | | 0.04 | 586.9 | 2.8 | | | 0.005 |
| Mode [PC 3] | | <.01 | | 0.03 | 561.4 | | | | 0.1 | 0.938 | | | -0.02 | | 0.03 | 556.4 | -0.7 | | | 0.505 |
| Pitch [PC 4] | | -0.02 | | 0.04 | 577.7 | | | | -0.6 | 0.571 | | | 0.04 | | 0.03 | 590.4 | 1.3 | | | 0.201 |
| Event Density [PC 5] | | 0.04 | | 0.04 | 564.5 | | | | 0.9 | 0.369 | | | 0.09 | | 0.04 | 610.6 | 2.4 | | | 0.017 |
| Tempo [PC 6] | | <.01 | | 0.03 | 563.3 | | | | 0.1 | 0.891 | | | 0.02 | | 0.03 | 588.1 | 0.7 | | | 0.461 |
| Pygmy Music [PM] | | -0.36 | | 0.11 | 550.4 | | | | -3.4 | 0.001 | | | -0.39 | | 0.1 | 592.2 | -4 | | | <.001 |
| Respiration Scores | | | | | | | | | | | | | | | | | | | | |
| [Int] | | -0.05 | | 0.08 | 161.8 | | | -0.6 | | 0.581 | | | 0.07 | | 0.08 | 178 | 1 | | | 0.338 |
| Roughnes/RMS Energy [PC 1] | | 0.04 | | 0.03 | 473.5 | | | 1.2 | | 0.229 | | | 0.09 | | 0.03 | 545.2 | 3.3 | | | 0.001 |
| Spectral Centroid [PC 2] | | 0.07 | | 0.04 | 467.5 | | | 1.7 | | 0.084 | | | 0.08 | | 0.03 | 527.4 | 2.3 | | | 0.02 |
| Mode [PC 3] | | -0.06 | | 0.03 | 472.3 | | | -2.1 | | 0.033 | | | -0.1 | | 0.03 | 516.5 | -3.9 | | | <.001 |
| Pitch [PC 4] | | -0.06 | | 0.03 | 476.3 | | | -1.7 | | 0.084 | | | -0.03 | | 0.03 | 536.2 | -1.2 | | | 0.231 |
| Event Density [PC 5] | | 0.04 | | 0.04 | 470.1 | | | 1 | | 0.315 | | | 0.08 | | 0.03 | 540.1 | 2.2 | | | 0.026 |
| Tempo [PC 6] | | 0.06 | | 0.03 | 475.5 | | | 2.1 | | 0.038 | | | 0.04 | | 0.03 | 531 | 1.5 | | | 0.139 |
| Pygmy Music [PM] | | 0.03 | | 0.09 | 459.2 | | | 0.3 | | 0.775 | | | -0.18 | | 0.08 | 532.3 | -2.1 | | | 0.039 |
| Valence Scores | | | | | | | | | | | | | | | | | | | | |
| [Int] | | -0.19 | | 0.06 | 619.1 | | | -3.2 | | 0.002 | | | -0.04 | | 0.06 | 631.2 | -0.7 | | | 0.511 |
| Roughnes/RMS Energy [PC 1] | | -0.03 | | 0.04 | 690.4 | | | -0.9 | | 0.357 | | | -0.1 | | 0.03 | 714.5 | -3.1 | | | 0.002 |
| Spectral Centroid [PC 2] | | -0.07 | | 0.05 | 697.9 | | | -1.4 | | 0.161 | | | -0.1 | | 0.04 | 706.4 | -2.4 | | | 0.018 |
| Mode [PC 3] | | -0.03 | | 0.04 | 702.3 | | | -0.9 | | 0.36 | | | 0.03 | | 0.03 | 652.6 | 1 | | | 0.312 |
| Pitch [PC 4] | | 0.03 | | 0.04 | 705.6 | | | 0.8 | | 0.405 | | | -0.24 | | 0.04 | 712.8 | -6.6 | | | <.001 |
| Event Density [PC 5] | | 0.02 | | 0.05 | 692 | | | 0.5 | | 0.642 | | | 0.3 | | 0.04 | 730.6 | 7.2 | | | <.001 |
| Tempo [PC 6] | | 0.03 | | 0.04 | 704.5 | | | 0.7 | | 0.494 | | | 0.1 | | 0.03 | 700.6 | 2.9 | | | 0.004 |
| Pygmy Music [PM] | | 0.46 | | 0.12 | 707.5 | | | 3.8 | | <.001 | | | 0.18 | | 0.11 | 712.2 | 1.6 | | | 0.104 |

*Note:* [Int] = Intercept.
